# Protein Kinase SGK2 Is Induced by the β_3_ Adrenergic Receptor-cAMP-PKA-PGC-1α/NT-PGC-1α Axis but Dispensable for Brown/Beige Adipose Tissue Thermogenesis

**DOI:** 10.3389/fphys.2021.780312

**Published:** 2021-11-25

**Authors:** Chul-Hong Park, Jiyoung Moon, Minsung Park, Helia Cheng, Jisu Lee, Ji Suk Chang

**Affiliations:** Gene Regulation and Metabolism Laboratory, Pennington Biomedical Research Center, Baton Rouge, LA, United States

**Keywords:** brown adipocytes, beige adipocytes, beta-adrenergic receptor, thermogenesis, SGK2 kinase, PPARGC1A

## Abstract

Brown and beige adipocytes are specialized to dissipate energy as heat. *Sgk2*, encoding a serine/threonine kinase, has been identified as a brown and beige adipocyte-specific gene in rodents and humans; however, its function in brown/beige adipocytes remains unraveled. Here, we examined the regulation and role of *Sgk2* in brown/beige adipose tissue thermogenesis. We found that transcriptional coactivators PGC-1α and NT-PGC-1α activated by the β_3_ adrenergic receptor-cAMP-PKA pathway are recruited to the *Sgk2* promoter, triggering *Sgk2* transcription in response to cold. SGK2 elevation was closely associated with increased serine/threonine phosphorylation of proteins carrying the consensus RxRxxS/T phosphorylation site. However, despite cold-dependent activation of SGK2, mice lacking *Sgk2* exhibited normal cold tolerance at 4°C. In addition, *Sgk2^+/+^* and *Sgk2^−/−^* mice induced comparable increases in energy expenditure during pharmacological activation of brown and beige adipose tissue with a β_3_AR agonist. *In vitro* loss- and gain-of-function studies further demonstrated that Sgk2 ablation or activation does not alter thermogenic gene expression and mitochondrial respiration in brown adipocytes. Collectively, our results reveal a new signaling component SGK2, although dispensable for cold-induced thermogenesis that adds an additional layer of complexity to the β_3_AR signaling network in brown/beige adipose tissue.

## Introduction

While white adipocytes store energy as triglycerides, brown adipocytes located in interscapular brown adipose tissue (BAT) transform the nutrient-derived chemical energy into heat through thermogenic respiration, which requires uncoupling protein 1 (UCP1) in the mitochondria (Golozoubova et al., 2001; Nedergaard et al., 2001; Cannon and Nedergaard, 2004). Beige adipocytes, an inducible form of thermogenic adipocytes, also emerge within subcutaneous white adipose tissue (WAT) after prolonged exposure to cold or β_3_-adrenergic receptor (β_3_AR) agonists (Granneman et al., 2005; Wu et al., 2012). The presence of brown and beige adipocytes in humans has also been established, and their stimulation by cold or β_3_AR agonists is associated with increased energy expenditure and enhanced disposal of circulating glucose and fatty acids, thus making them an attractive target for the treatment of obesity and diabetes (Cypess et al., 2009, 2013, 2015; van Marken Lichtenbelt et al., 2009; Blondin et al., 2020; O’Mara et al., 2020).

Brown and beige adipocytes show similar morphology and function with high expression of several markers such as *Ucp1*, *Cidea*, *Dio2*, *Elovl3*, *Cox7a1*, *Ppargc1a*, and *Gyk* (Himms-Hagen et al., 1994; Wu et al., 2012). Recently, *Sgk2*, serum-, and glucocorticoid-inducible kinase 2, has been identified as an additional gene preferentially expressed in brown and beige adipocytes (Harms et al., 2014). *Sgk2* gene expression is markedly elevated by cold in murine brown and beige adipocytes compared to white adipocytes (Rosell et al., 2014; Perdikari et al., 2018) and its transcripts are also enriched in human supraclavicular BAT compared to subcutaneous WAT (Perdikari et al., 2018; Toth et al., 2020). SGK2 belongs to the SGK serine/threonine kinase family that is highly homologous to the AKT kinase family (Pearce et al., 2010). Both SGK and AKT are activated by signals stimulating phosphatidylinositol 3-kinase (PI3K) and phosphorylate serine and threonine residues that lie within the consensus RxRxxS/T motifs (Alessi et al., 1996; Kobayashi et al., 1999; Manning and Cantley, 2007; Hemmings and Restuccia, 2012). Although they have overlapping substrates (Brunet et al., 2001; Sakoda et al., 2003; Lee et al., 2007), a growing body of evidence indicates that SGK and AKT are activated under distinct physiological cues, phosphorylate distinct proteins, and have different functions (Sakoda et al., 2003; Lang et al., 2006; Toker and Marmiroli, 2014). Several studies reported that SGK2, like the most studied isoform SGK1, modulates the function of membrane proteins such as Na^+^/H^+^ exchanger (Pao et al., 2010), organic anion transporter (Wang et al., 2016; Xu et al., 2016), and Na^+^ channel (Friedrich et al., 2003) in kidney proximal tubule cells. Recent works also indicate that SGK2 plays a role in autophagy (Ranzuglia et al., 2020) and cancer biology supporting tumor progression (Chen et al., 2018; Liu et al., 2019). However, despite selective expression of SGK2 in brown and beige adipocytes compared to white adipocytes, its function in brown and beige adipocyte thermogenesis has not been examined to date.

In the present study, we aimed to investigate the mechanism by which *Sgk2* gene expression is upregulated in brown and beige adipocytes and the importance of SGK2 signaling in adaptive thermogenesis during cold stress or β_3_AR stimulation.

## Materials and Methods

### Animal Studies

C57BL/6 mice were purchased from Jackson laboratory. *Sgk2^em1(IMPC)Mbp^* mice containing heterozygous deletion of the *Sgk2* exon 4 were purchased from the Mutant Mouse Regional Resource Centers (MMRRC). The heterozygotes were mated to obtain homozygous *Sgk2^−/−^* mice and littermate *Sgk2^+/+^* control mice. Genotyping was performed by PCR using ear punch DNA. All mice were housed in standard conditions (22–23°C; 12-h light/12-h dark cycle) and maintained on a regular chow diet (5,001, LabDiet, St. Louis, MO) with *ad libitum* feeding.

For cohort 1, 9-week-old C57BL/6 female mice were randomly assigned to three groups and singly housed at near thermoneutrality (28°C; *n*=10) or exposed to 4°C for 5h (*n*=10) and 7days (*n*=7). For cohort 2, 8-week-old C57BL/6 male mice were randomly assigned to three groups and administered intraperitoneally with vehicle (*n*=8) or a β_3_-adrenergic receptor agonist CL316243 (1mg/kg body weight/day) for 5h (*n*=8) and 7days (*n*=5) during single-housing at 28°C. For cohort 3, 7-week-old *Sgk2^+/+^* and *Sgk2^−/−^* female mice were singly housed at 28°C or exposed to 4°C for 9days (*n*=6–8 per group). Core rectal temperature was measured at baseline and every 1h over the 8h-period of cold exposure. For cohort 4, 12-week-old *Sgk2^+/+^* and *Sgk2^−/−^* male mice (*n*=8 per genotype) were weighed and their body composition was measured using a Bruker Minispec Mouse Analyzer (Bruker Optics, Billerica, MA, United States). Mice were then placed in indirect calorimetry chambers (Sable Systems International, North Las Vegas, NV) and monitored for VO_2_ and VCO_2_ at 28°C. After 2days in chambers, mice were intraperitoneally injected with CL316243 (1mg/kg body weight/day) for 4days and continuously monitored for VO_2_ and VCO_2_. After removing from the chambers, mice were injected with CL316243 for additional 6days.

At the end of experiments, mice from cohorts 1–4 were euthanized to collect brown and inguinal white adipose tissue by carbon dioxide asphyxiation followed by cervical dislocation that is in accordance with the established recommendations of the American Veterinary Medical Association (AVMA) Guidelines for the Euthanasia of Animals. All animal experimental procedures were approved by the Institutional Animal Care and Use Committee of the Pennington Biomedical Research Center and animal study reporting adheres to the ARRIVE guidelines (Kilkenny et al., 2010).

### Chromatin Immunoprecipitation Assay

Chromatin immunoprecipitation (ChIP) was performed using brown adipose tissue extracted from mice exposed to 4°C for 5h, as described previously (Chang et al., 2012, 2018). The cross-linked nuclear lysates were immunoprecipitated with PGC-1α antibody detecting both PGC-1α and NT-PGC-1α or rabbit IgG. PCR was carried out to examine the binding of PGC-1α/NT-PGC-1α to the ERRE region of the *Sgk2* promoter using following primers: 5'-CTATGGAAAGGGGGTGATTT (fwd), 5'-GGACCTTCCGGTTACTCATT (rev).

### Brown and Beige Adipocyte Differentiation

For brown adipocytes, interscapular brown adipose tissue was dissected from 4-day-old mice and digested by collagenase type I. Stromal vascular fraction (SVF) cells were collected by centrifugation at 700× *g* for 10min and cell suspension was filtered through a 50–70μm cell strainer. After centrifugation, cells were resuspended and seeded in complete DMEM medium, followed by immortalization with the retrovirus expressing SV40T antigen as described previously (Uldry et al., 2006; Zhang et al., 2009). After selection with 1μg/ml of puromycin, the immortalized brown preadipocytes were grown to confluence in complete DMEM medium and incubated for 48h in induction medium containing 20nM insulin, 1nMT3, 0.5mM isobutylmethylxanthine, 0.5μM dexamethasone, and 0.125mM indomethacin (Chang et al., 2010; Jun et al., 2014). Thereafter, the cells were maintained in differentiation medium containing 20nM insulin and 1nMT3 until day 7.

For beige adipocytes, the subcutaneous inguinal fat pad was dissected from 5-week-old mice and it was minced and digested with collagenase D and diapase II. SVF cells were then isolated as described above and previously (Aune et al., 2013). SVF cells were grown to confluence in complete DMEM medium and incubated for 48h in induction medium containing 5μg/ml insulin, 1nMT3, 0.5mM isobutylmethylxanthine, 5μM dexamethasone, 0.125mM indomethacin, and 0.5μM rosiglitazone, as described previously (Aune et al., 2013). Thereafter, cells were maintained in differentiation medium containing 5μg/ml insulin and 1nMT3 with 0.5μM rosiglitazone for 2days and 1μM rosiglitazone for 4days.

### Retrovirus Production and Infection

A retroviral pBABE-*Sgk2*-S356D plasmid was generated by subcloning a BamHI/XhoI-fragment of pcDNA3.1-*Sgk2*-S356D (Pao et al., 2010) into the BamHI/SalI sites of pBABE-neo (Addgene, Watertown, MA). Retrovirus expressing *Sgk2*-S356D was produced from GP-293 cells by co-transfecting pBABE-*Sgk2*-S356D with pVSV-G as described previously (Chang et al., 2010). Immortalized brown preadipocytes were then infected in retrovirus-containing medium supplemented with 8μg/ml of polybrene for 8h. After 48h, neomycin-resistant clones were selected and pooled.

### Cellular O_2_ Consumption Rates

Oxygen consumption rates (OCR) of differentiated brown adipocytes were monitored using an Oxygraph-2k (Oroboros Instruments, Innsbruck, Austria) as described previously (Jun et al., 2014). Briefly, cells were placed in a magnetically stirred respirometric chamber containing the culture medium. OCR measurements were obtained at baseline and after injection of oligomycin, FCCP and antimycin A. The value of basal, leak, and maximal mitochondrial respiration was determined by subtracting non-mitochondrial respiration as described in the Oroboros Operator’s Manual.

### Western Blot Analysis

Whole-cell extracts were prepared from tissues or cells by homogenization in lysis buffer (Chang et al., 2010) and subjected to Western blot analysis using the following antibodies: anti-SGK2 (#5595), anti-phospho-AKT S473 (#9271), anti-AKT (#9272), anti-phospho-RxRxxS/T (#10001), anti-phospho-GSK3α/β (#9331), anti-GSK3α/β (#5676; Cell Signaling, Danvers, MA), and anti-β actin (Sigma, St. Louis, MO).

### Quantitative Real-Time PCR Analysis

Total RNA from tissues or cells was reverse-transcribed for quantitative real-time PCR analysis as described previously (Chang et al., 2010, 2012). Gene expression analysis was carried out using the Applied Biosystems 7900 (Applied Biosystems) and iTaq Universal SYBR Green Supermix (Bio-Rad). Relative mRNA expression of the genes of interest was determined using gene-specific primers after normalization to cyclophilin by the 2^-ΔΔCt^ method. Primer sequences were obtained from the PrimerBank public resource (Wang and Seed, 2003). *Sgk2* fwd: 5'- CCAATGGGAACATCAACC-3'; *Sgk2* rev: 5'-CAGTAGGACCTTCCCGTAGT-3'.

### Statistical Analysis

All line and bar graphs were created by using the Prism 6 software (GraphPad Software, San Diego, CA, United States) and student *t* test or two-way ANOVA was used to compare the differences between groups using the Prism 6 software. Data are presented as mean±SEM. Values of *p*<0.05 were considered statistically significant.

## Results

### *Sgk2* Gene Expression Is Elevated by Cold-Stimulated β-Adrenergic Signaling in Brown and Beige Adipose Tissue

**Figure 1:**
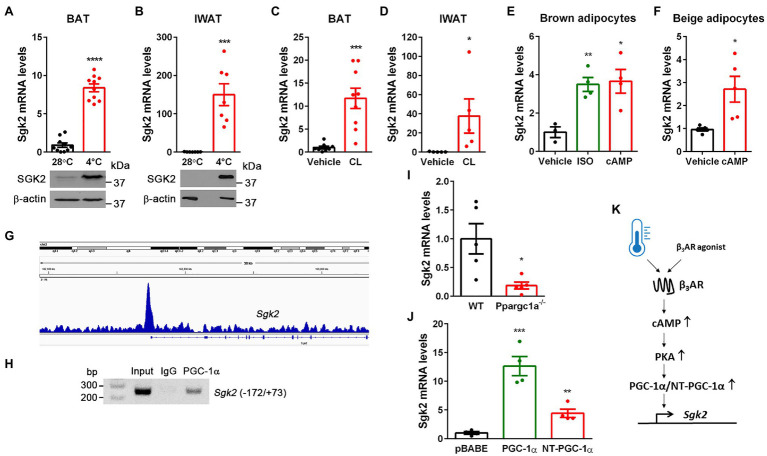
Cold- and CL316243-dependent upregulation of *Sgk2* gene expression is mediated by PGC-1α and NT-PGC-1α. **(A)** Effect of cold on *Sgk2* mRNA and protein levels in BAT. 9-week-old BL6 female mice were housed at 28°C (*n*=10) or exposed to 4°C for 5h (*n*=10). **(B)** Effect of cold on *Sgk2* mRNA and protein levels in IWAT. 9-week-old BL6 female mice were housed at 28°C (*n*=7) or exposed to 4°C for 7days (*n*=7). **(C)** Effect of β_3_AR stimulation on *Sgk2* expression in BAT. 8-week-old BL6 male mice were administered with vehicle (*n*=8) or a β_3_AR agonist CL316243 (*n*=8) for 5h. **(D)** Effect of β_3_AR stimulation on *Sgk2* expression in IWAT. 8-week-old BL6 male mice were administered with vehicle (*n*=5) or CL316243 (*n*=5) for 7days. **(E)** Upregulation of *Sgk2* expression by a βAR agonist or cAMP in brown adipocytes. Differentiated brown adipocytes (7days) were treated with vehicle, isoproterenol (5μM) or dibutyryl cAMP (0.5mM) for 4h (*n*=4 per group). **(F)** Upregulation of *Sgk2* expression by cAMP in primary beige adipocytes. Differentiated beige adipocytes (7days) were treated with vehicle or dibutyryl cAMP (0.5mM) for 4h (*n*=5 per group). **(G)** Enrichment of PGC-1α/NT-PGC-1α on the *Sgk2* promoter in cold-activated BAT. The PGC-1α/NT-PGC-1α ChIP-seq peak (Chang et al., 2018) was visualized by the Integrative Genomics Viewer (IGV) v2.3. **(H)** PCR analysis of PGC-1α/NT-PGC-1α recruitment to the ERRE of the *Sgk2* promoter. ChIP was carried out with PGC-1α antibody (Chang et al., 2018) in nuclear extracts of BAT isolated from mice exposed to 4°C for 5h. **(I)** Decreased induction of *Sgk2* expression in *Ppargc1a* ^−/−^ brown adipocytes treated with dibutyryl cAMP (*n*=5 per group). **(J)** PGC-1α- and NT-PGC-1α-dependent upregulation of *Sgk2* expression in *Ppargc1a* ^−/−^ brown adipocytes (*n*=4 per group). **(K)** Schematic presentation of transcriptional regulation of *Sgk2* by cold or β_3_AR agonist. All data are presented as the mean±SEM. ^*^p<0.05, ^**^p<0.01, ^***^p<0.001, ^****^p<0.0001 determined by Student’s *t* test.

Previous genome-wide transcriptome analyses revealed enrichment of *Sgk2* transcripts in cold-activated brown and beige adipocytes compared to white adipocytes in rodents and humans (Rosell et al., 2014; Perdikari et al., 2018; Toth et al., 2020). Indeed, acute cold exposure significantly elevated *Sgk2* mRNA and protein levels in BAT (Figure 1A). Similarly, the *Sgk2* mRNA and protein levels were markedly induced in inguinal WAT undergoing browning during prolonged cold exposure, although they are barely detectable in IWAT of mice housed at 28°C (Figure 1B). In addition, pharmacological stimulation of BAT and IWAT by a β_3_AR agonist CL316243 (Himms-Hagen et al., 1994, 2000; Granneman et al., 2005; Chang et al., 2012) mimicked the effect of cold on *Sgk2* gene expression (Figures 1C,D). To further confirm the direct effect of βAR signaling on *Sgk2* gene expression in brown and beige adipocytes, we differentiated brown preadipocytes (Uldry et al., 2006; Jun et al., 2014) into brown adipocytes and treated with a βAR agonist isoproterenol or a cell-permeable cAMP analog dibutyryl cAMP, which mimics the main intracellular regulatory mechanism activated by βAR stimulation. In line with *in vivo* data, isoproterenol and dibutyryl cAMP significantly increased *Sgk2* gene expression in brown adipocytes (Figure 1E). Similarly, differentiation of stromal vascular cells isolated from IWAT into beige adipocytes and subsequent treatment with dibutyryl cAMP increased *Sgk2* gene expression in beige adipocytes (Figure 1F).

### Cold-Induced Transcriptional Coactivators, PGC-1α and NT-PGC-1α, Promote *Sgk2* Gene Expression

Stimulation of βAR in brown adipocytes signals through coupling to G-proteins, adenylyl cyclase, and cAMP-dependent protein kinase A (PKA), which in turn activates CREB transcription factor, leading to increased *Ppargc1a* gene expression (Cannon and Nedergaard, 2004). We previously showed that the *Ppargc1a* gene produces a full-length PGC-1α and a shorter isoform NT-PGC-1α that are key transcriptional regulators of cold-induced thermogenesis in BAT (Zhang et al., 2009; Chang et al., 2010, 2012; Chang and Ha, 2017). Interestingly, our genome-wide analysis of PGC-1α/NT-PGC-1α binding in BAT by chromatin immunoprecipitation sequencing (ChIP-seq; Chang et al., 2018) revealed enrichment of PGC-1α/NT-PGC-1α on the *Sgk2* gene promoter that contains an estrogen-related receptor (ERR) response element (ERRE; Figure 1G). To confirm this finding, we carried an independent ChIP assay with PGC-1α antibody recognizing both PGC-1α and NT-PGC-1α (Chang et al., 2018). Indeed, PGC-1α/NT-PGC-1α were recruited to the ERRE region of the *Sgk2* gene promoter in cold-activated BAT (Figure 1H).

Next, to examine whether PGC-1α and/or NT-PGC-1α regulate *Sgk2* gene expression, we used loss- and gain-of-function approaches. Ablation of both PGC-1α and NT-PGC-1α (*Ppargc1a* ^−/−^) in brown adipocytes blunted *Sgk2* gene expression (Uldry et al., 2006; Jun et al., 2014; Figure 1I). Conversely, expression of either PGC-1α or NT-PGC-1α in *Ppargc1a* ^−/−^ brown adipocytes efficiently restored *Sgk2* gene expression with a more pronounced effect by PGC-1α (Figure 1J). Taken together, these results clearly demonstrate that *Sgk2* gene expression is upregulated by the well-established β_3_AR-cAMP-PKA-PGC-1α/NT-PGC-1α pathway (Figure 1K).

### SGK2 Activation Induces Phosphorylation of Its Downstream Substrates Containing RxRxxS/T Motifs in Brown Adipocytes but Is Not Sufficient to Enhance Thermogenesis

**Figure 2:**
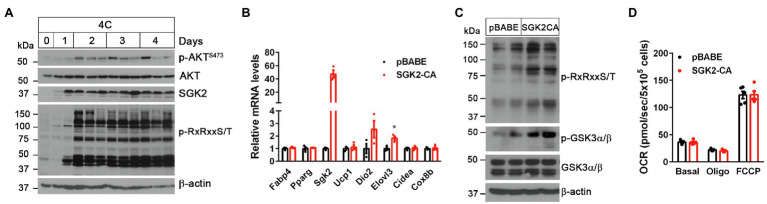
SGK2 activation induces phosphorylation of downstream substrates in brown adipocytes. **(A)** Cold-dependent activation of SGK2 signaling in BAT. 9-week old BL6 mice were exposed to 4°C for indicated times. **(B)** No change in expression of genes involved in brown adipogenesis and thermogenesis by SGK2 activation. Brown preadipocytes were transduced with retrovirus expressing an empty vector or SGK2-S356D, followed by differentiation into brown adipocytes for 7days (*n*=3 per group). **(C)** Effect of SGK2 activation on downstream signaling in brown adipocytes starved for serum for 8h. **(D)** Effect of SGK2 activation on mitochondrial respiration in brown adipocytes. Oxygen consumption rates (OCR) were measured in differentiated brown adipocytes (7days) at baseline and after addition of oligomycin and FCCP (*n*=5 per group). All data are presented as the mean ± SEM. ^*^p < 0.05 determined by Student’s *t* test.

SGK2 belongs to the SGK kinase family that is closely related to the AKT serine/threonine kinase (Pearce et al., 2010). Both SGK and ATK phosphorylate serine and threonine residues that lie within the consensus RxRxxS/T motifs (Alessi et al., 1996; Kobayashi et al., 1999; Manning and Cantley, 2007; Hemmings and Restuccia, 2012). During cold stress, AKT protein levels remained unchanged in BAT but its activity increased by cold, as reflected by mTORC2-mediated phosphorylation of AKT on Ser473 in the C-terminal hydrophobic motif (Sarbassov et al., 2005; Albert et al., 2016; Figure 2A). In contrast to AKT, SGK2 protein levels were markedly elevated by cold in BAT. We were not able to assess its activity due to lack of SGK2 antibody detecting phosphorylation on Ser356 that is equivalent to the C-terminal phosphorylation site of AKT (Kobayashi et al., 1999; Pao et al., 2010). However, cold-induced phosphorylation of proteins carrying RxRxxS/T motifs was more closely associated with SGK2 protein levels rather than AKT activity (Figure 2A), suggesting that cold-induced SGK2 is an active serine/threonine kinase in BAT.

To test if SGK2 phosphorylates its downstream targets in brown adipocytes, we expressed a constitutively active form of SGK2 (SGK2-S356D; Kobayashi et al., 1999; Pao et al., 2010) in brown adipocytes and examined changes in RxRxxS/T phosphorylation status in the absence of βAR stimulation. SGK2 activation did not alter brown adipogenesis, as evidenced by comparable expression of adipogenic marker genes (*Fabp4* and *Ppparg*) and brown adipocyte-enriched genes (*Ucp1, Dio2, Elovl3, Cidea*, *and Cox8b*) between the groups (Figure 2B). As expected, SGK2-CA led to increased phosphorylation of its substrates carrying RxRxxS/T motifs in the absence of βAR signaling (Figure 2C). A recent study reported that cold exposure and β-adrenergic stimulation cause phosphorylation of glycogen synthase kinase 3 (GSK3), a multifunctional serine/threonine kinase, in a PKA-dependent manner, leading to inhibition of its negative effect on the MKK3/6-p38 MAPK-ATF2 signaling pathway downstream of β_3_AR (Markussen et al., 2018). Given that GSK3 isoforms α and β are the well-known AKT/SGK targets (Cross et al., 1995; Sakoda et al., 2003), we examined whether SGK2-CA phosphorylates GSK3α and GSK3β on serine residues in RARTTS^21^ and RPRTTS^9^, respectively (Cross et al., 1995; Sakoda et al., 2003), in brown adipocytes. Indeed, SGK2-CA increased phosphorylation of GSK3 with a more pronounced effect on GSK3α (51kDa; Figure 2C), suggesting that SGK2 induced by the βAR-PKA-PGC-1α/NT-PGC-1α pathway could participate in phosphorylation and inhibition of GSK3α, which is a negative regulator of βAR signaling in BAT.

Next, we examined whether SGK2 activation enhances thermogenic activity in brown adipocytes by measuring mitochondrial respiration. Mitochondrial respiration by the electron transport chain (ETC) is critical for UCP1-mediated thermogenesis (Golozoubova et al., 2001; Nedergaard et al., 2001). The ETC creates a proton gradient across the inner mitochondrial membrane and UCP1 subsequently allows protons to return to the mitochondrial matrix, resulting in heat production (Golozoubova et al., 2001; Nedergaard et al., 2001). Despite increased phosphorylation of its downstream substrates including GSK3, SGK2 activation did not lead to an increase in mitochondrial respiration (Figure 2D). Oligomycin-insensitive leak respiration, which in part represents UCP1-mediated thermogenesis, and FCCP-induced maximum respiration were also comparable between the groups. It is likely that GSK3 inhibition itself by SGK2, without activation of βAR signaling, has no effect on thermogenic activity. Thus, these results indicate that activation of SGK2 alone is not sufficient to promote brown adipocyte thermogenesis.

### SGK2 Is Dispensable for Cold- and β_3_AR Agonist-Stimulated Thermogenesis

**Figure 3:**
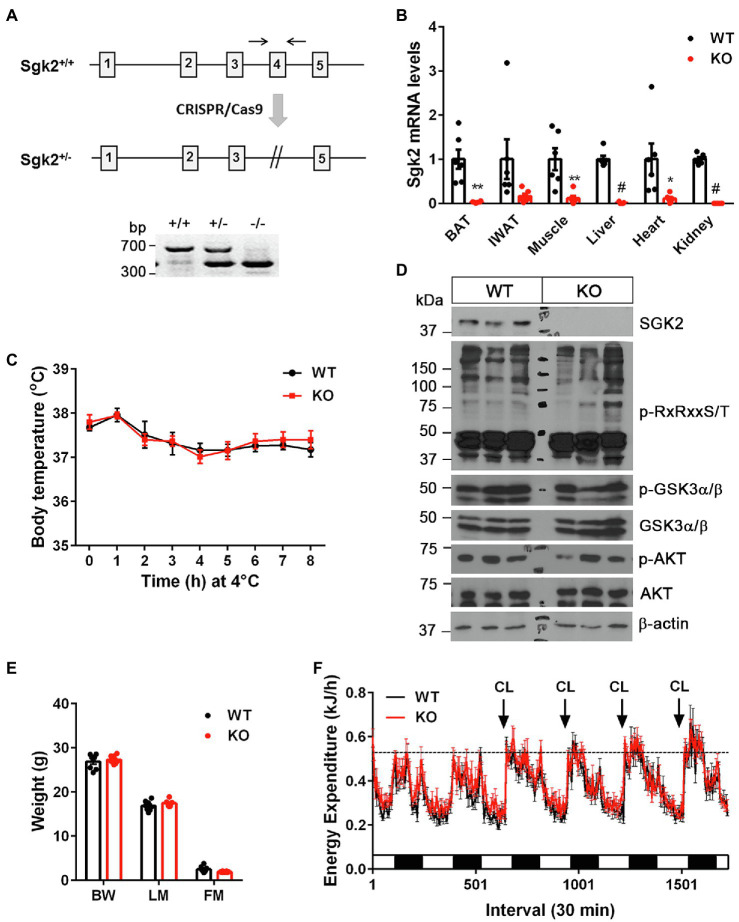
Sgk2 ablation had no effect on cold- and CL316243-stimulated thermogenesis. **(A)** Generation of *Sgk2* knockout mice. Top panel, a region of the mouse *Sgk2* gene containing exons 1–5. The exon 4 was targeted for deletion by using the CRISPR/Cas9 system. Bottom panel, PCR analysis of ear punch samples from *Sgk2^+/+^*, *Sgk2^+/−^*, and *Sgk2^−/−^* mice using a primer pair described as two arrows. **(B)** Quantitative real-time PCR analysis for detection of *Sgk2* transcripts in *Sgk2^+/+^* (WT) and *Sgk2^−/−^* (KO) female mice (*n*=6 per group). For IWAT, tissue samples were dissected from mice exposed to 4°C for 9days (*n*=6 per group). Data are presented as the mean±SEM. ^*^p<0.05, ^**^p<0.01, ^#^*p*<0.0001 determined by Student’s *t* test. **(C)** Body temperature of 7-week-old *Sgk2^+/+^* (*n*=8) and *Sgk2^−/−^* (*n*=6) female mice during 8h of cold exposure. Core body temperature were measured using a rectal thermometer at indicated times. **(D)** Western blot analysis of BAT dissected from 8-week-old *Sgk2^+/+^* and *Sgk2^−/−^* female mice exposed to 4°C for 9days. **(E)** Body weight (BW), lean mass (LM) and fat mass (FM) of 12-week-old *Sgk2^+/+^* (*n*=8) and *Sgk2^−/−^* (*n*=8) male mice. **(F)** Energy expenditure of 12-week-old *Sgk2^+/+^* (*n*=8) and *Sgk2^−/−^* (*n*=8) male mice prior to and during administration of a β_3_AR agonist CL316243.

To investigate whether cold-induced SGK2 is required for cold-stimulated thermogenesis, we generated *Sgk2^−/−^* mice by mating heterozygous *Sgk2^em1(IMPC)Mbp^* mice containing deletion of the *Sgk2* exon 4. The phenotype of *Sgk2^−/−^* mice has not been characterized to date. The mutant allele was confirmed by PCR analysis of genomic DNA isolated form *Sgk2^+/+^*, *Sgk2^+/−^*, and *Sgk2^−/−^* mice (Figure 3A). The efficacy of gene targeting was further examined by qPCR analysis. As expected, *Sgk2* transcripts were absent in all tissues including BAT, IWAT, muscle, liver, heart and kidney of *Sgk2^−/−^* mice (Figure 3B), clearly demonstrating the loss of *Sgk2*. Next, we exposed *Sgk2^+/+^* and *Sgk2^−/−^* female mice to 4°C for 8h and measured core body temperature to determine the effect of *Sgk2* ablation on cold-induced thermogenesis. Despite marked elevation of SGK2 by cold, mice lacking *Sgk2* were able to maintain body temperature during cold exposure (Figure 3C). Further analysis of cold-activated BAT revealed no changes in phosphorylation status of RxRxxS/T-containing proteins by *Sgk2* ablation (Figure 3D). In addition, phosphorylation levels of GSK3α on Ser21 were relatively comparable in *Sgk2^+/+^* and *Sgk2^−/−^* BAT, implying that other kinases are able to replace SGK2 function in cold-activated BAT.

To rule out the possibility that increased muscle shivering contributes to the thermoregulation of *Sgk2^−/−^* mice during cold exposure, we placed *Sgk2^+/+^* and *Sgk2^−/−^* male mice in indirect calorimetric chambers at thermoneutral temperature (28°C) and measured energy expenditure during pharmacological stimulation of brown and beige adipose tissue with a highly selective β_3_AR agonist CL316243. CL316243-dependent increases in energy expenditure represent brown and beige adipose thermogenesis (Himms-Hagen et al., 1994, 2000; Granneman et al., 2005; Chang et al., 2012). At 12weeks of age, *Sgk2^+/+^* and *Sgk2^−/−^* mice exhibited similar body weights and composition (Figure 3E). In addition, energy expenditure was comparable between *Sgk2^+/+^* and *Sgk2^−/−^* mice prior to introduction of the agonist (Figure 3F). Daily administration of CL316243 for 4days produced comparable increases in energy expenditure in *Sgk2^+/+^* and *Sgk2^−/−^* mice (Figure 3F), indicating that SGK2 is not required for CL316243-stimulated thermogenesis in brown and beige adipose tissue. The thermogenic response of *Sgk2^−/−^* mice to physiological (cold) or pharmacological (β_3_AR agonist) stimulation was same regardless of the distinct use of sex of mice (Figures 3C,F). Locomotor activity and food intake over the 6-day period did not differ between the genotypes (Supplementary Figures 1A,B). It is well documented that CL316243-mediated activation of brown and beige adipose tissue upregulates genes involved in mitochondrial biogenesis, thermogenesis, mitochondrial electron transport activity, fatty acid oxidation, lipid metabolism, and glucose metabolism (Yu et al., 2002; Chang et al., 2012; Mottillo et al., 2014; Hao et al., 2015; Kim et al., 2018). Thus, we examined the gene expression profiles of BAT and IWAT from *Sgk2^+/+^* and *Sgk2^−/−^* mice treated with CL316243 for 10days. In line with energy expenditure results, CL316243-induced gene expression was comparable in *Sgk2^+/+^* and *Sgk2^−/−^* mice (Supplementary Figures 1C,D), demonstrating that SGK2 signaling is dispensable for CL316243-induced remodeling of brown and beige adipose tissue.

### Loss of SGK2 Activity in Brown Adipocytes Has No Effect on Mitochondrial Respiration and Thermogenesis

**Figure 4:**
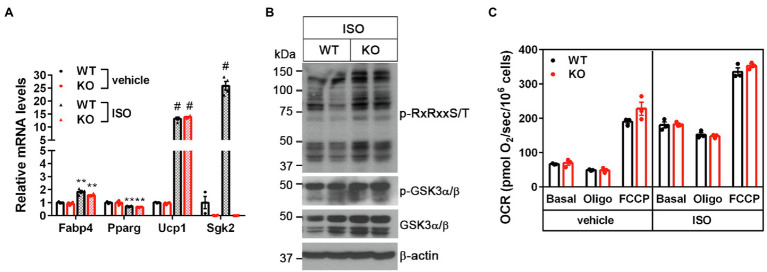
*Sgk2* ablation does not alter βAR-stimulated mitochondrial respiration in brown adipocytes. **(A)** No change in brown adipogenesis by *Sgk2* ablation. *Sgk2^+/+^* (WT) and *Sgk2^−/−^* (KO) brown preadipocytes were differentiated into brown adipocytes for 7days and treated with or without isoproterenol for 4h (*n*=3–4 per group). **(B)** Effect of *Sgk2* ablation on downstream signaling in isoproterenol-stimulated brown adipocytes. **(C)** Effect of *Sgk2* ablation on mitochondrial respiration in differentiated brown adipocytes (7days) in the absence and presence of βAR stimulation. Oxygen consumption rates (OCR) were measured at baseline and after addition of oligomycin and FCCP (*n*=4 per group). All data are presented as the mean±SEM. ^**^p<0.01, ^#^*p*<0.0001 determined by Student’s *t* test.

To further determine the cell-autonomous effect of *Sgk2* ablation in brown adipocytes, we isolated stromal vascular fraction (SVF) cells from BAT of *Sgk2^+/+^* and *Sgk2^−/−^* mice and induced differentiation of immortalized brown preadipocytes into brown adipocytes. Brown adipogenesis was not affected by *Sgk2* ablation, as evidenced by similar mRNA expression of *Fabp4*, *Pparg*, and *Ucp1* in *Sgk2^+/+^* and *Sgk2^−/−^* brown adipocytes (Figure 4A). *Sgk2* ablation in brown adipocytes did not lead to decreased serine/threonine phosphorylation of RxRxxS/T-containing proteins in response to βAR stimulation (Figure 4B). Rather, serine/threonine phosphorylation levels were slightly higher in *Sgk2^−/−^* brown adipocytes (Figure 4B). In addition, GSK3α phosphorylation levels were not altered by *Sgk2* ablation. Next, we assessed thermogenic activity of *Sgk2^+/+^* and *Sgk2^−/−^* brown adipocytes by measuring isoproterenol-stimulated mitochondrial respiration. Isoproterenol produced a comparable increase in mitochondrial respiration in *Sgk2^+/+^* and *Sgk2^−/−^* brown adipocytes (Figure 4C). Leak respiration, which in part represents UCP1-mediated thermogenesis, and FCCP-induced maximum respiration were also comparable in *Sgk2^+/+^* and *Sgk2^−/−^* brown adipocytes. Taken together, these results demonstrate that SGK2 activity is dispensable for βAR-stimulated mitochondrial respiration and thermogenesis. Given the same effect of *Sgk2* ablation on thermogenesis in mice and cells, it is not likely that immortalization is affecting the data on the role of *Sgk2* in brown adipocytes.

## Discussion

*Sgk2* is a BAT-enriched gene that is highly expressed in cold-activated brown and beige adipocytes in rodents and humans (Harms et al., 2014; Rosell et al., 2014; Perdikari et al., 2018; Toth et al., 2020). The present study clearly delineates that *Sgk2* gene expression is upregulated by PGC-1α and NT-PGC-1α that are recruited to the ERRE of the *Sgk2* promoter upon cold exposure. ERRα and ERRγ have been shown to bind on the *Sgk2* promoter, triggering its transcription in the kidney (Tremblay et al., 2010; Zhao et al., 2018). Given the ability of PGC-1α and NT-PGC-1α to coactivate ERRs in BAT (Huss et al., 2002; Lin et al., 2005; Chang et al., 2012), we postulate that cold-induced PGC-1α and NT-PGC-1α activate ERRs bound to the ERRE of the *Sgk2* promoter, leading to increased expression of *Sgk2* in cold-activated brown and beige adipose tissue.

Despite marked elevation of SGK2 in brown and beige adipose tissue by cold or β_3_AR agonists, mice lacking *Sgk2* showed the normal ability to increase brown and beige adipose thermogenesis during cold exposure or β_3_AR stimulation. *In vitro* loss- and gain-of-function studies further demonstrated that *Sgk2* ablation or activation does not alter mitochondrial respiration and thermogenesis in brown adipocytes. These findings indicate that SGK2 signaling is not directly involved in promoting brown/beige adipose thermogenesis. However, GSK3 phosphorylation by SGK2 in part suggests its indirect role in regulating brown/beige adipose thermogenesis. Phosphorylation of GSK3 by cold or β_3_AR stimulation has been shown to inhibit its negative effect on the MKK3/6-p38 MAPK-ATF2 signaling pathway downstream of β_3_AR, leading to enhanced thermogenic gene expression in BAT (Markussen et al., 2018). Thus, cold-induced SGK2 may participate in the suppression of GSK3 activity, along with AKT (Cross et al., 1995) and PKA (Fang et al., 2000), although its contribution seems small because *Sgk2* ablation resulted in no change in GSK3 phosphorylation levels in cold-activated BAT.

Several studies reported that Na^+^ influx is increased in brown adipocytes during the norepinephrine/βAR-stimulated depolarization (Girardier and Schneider-Picard, 1983; Connolly et al., 1986) although its physiological significance remains to be elucidated. Given the role of SGK2 in modulating Na^+^ channels in kidney cells (Friedrich et al., 2003; Pao et al., 2010; Wang et al., 2016; Xu et al., 2016), it would be interesting to determine if SGK2 regulates Na^+^ influx in brown/beige adipocytes during the βAR-stimulated depolarization.

In summary, our findings illustrate a new signaling component, SGK2, that adds an additional layer of complexity to the β_3_AR signaling network in brown/beige adipose tissue although it is dispensable for cold-induced thermogenesis.

## Data Availability Statement

The datasets presented in this study can be found in online repositories. The names of the repository/repositories and accession number(s) can be found at: (NCBI)’s Gene Expression Omnibus (GEO) database (accession number GSE110056).

## Ethics Statement

The animal study was reviewed and approved by Institutional Animal Care and Use Committee of the Pennington Biomedical Research Center.

## Author Contributions

C-HP, JM, MP, HC, and JL carried out the experiments and analyzed the data. JSC conceived of the presented idea, analyzed the data, and wrote the manuscript. All authors contributed to the article and approved the submitted version.

## Funding

This work was partially supported by the National Institutes of Health grants NIH R01DK104748 (JSC) and COBRE (NIH8 1P30GM118430-01). This work used the Genomics Core and Cell Biology and Bioimaging Core that are supported in part by COBRE (NIH8 1P30GM118430-01) and NORC (NIH P30-DK072476) center grants from the National Institutes of Health.

## Conflict of Interest

The authors declare that the research was conducted in the absence of any commercial or financial relationships that could be construed as a potential conflict of interest.

## Publisher’s Note

All claims expressed in this article are solely those of the authors and do not necessarily represent those of their affiliated organizations, or those of the publisher, the editors and the reviewers. Any product that may be evaluated in this article, or claim that may be made by its manufacturer, is not guaranteed or endorsed by the publisher.
