# Supplementary material for: Protein Kinase SGK2 Is Induced by the β3 Adrenergic Receptor-cAMP-PKA-PGC-1α/NT-PGC-1α Axis but Dispensable for Brown/Beige Adipose Tissue Thermogenesis
Source: Front Physiol. 2021 Nov 25;12:780312. doi: 10.3389/fphys.2021.780312 (PMC8657153; doi:10.3389/fphys.2021.780312)
Supplement: Supplementary file 1 [file Image_1.pdf]

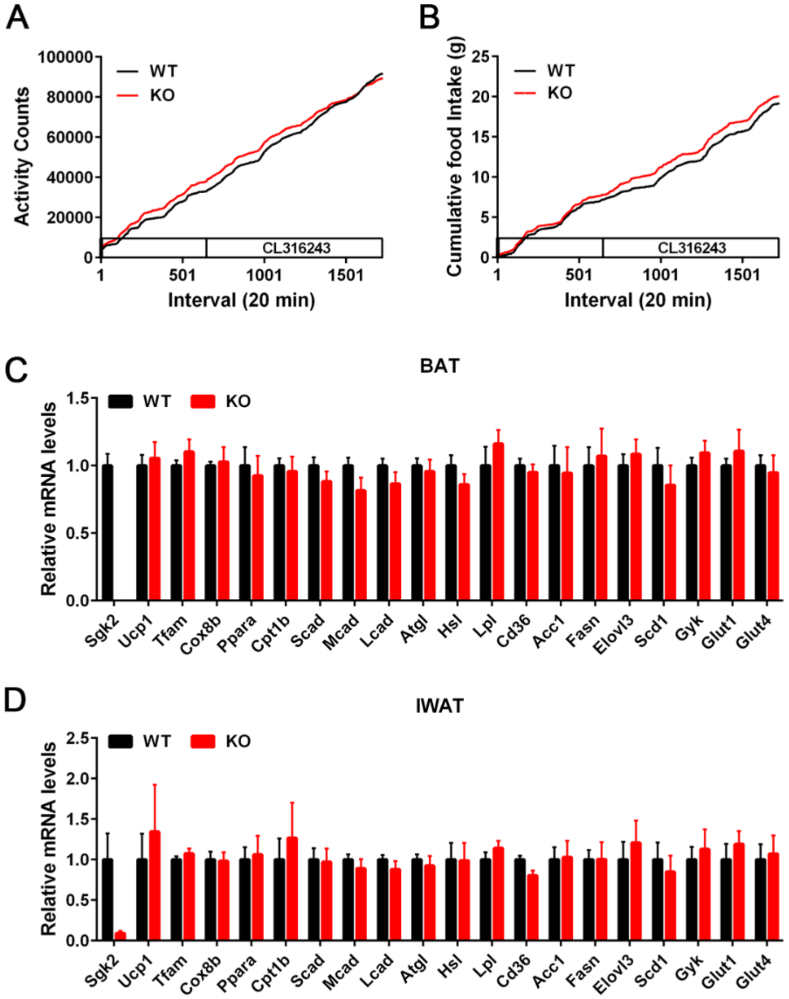

Supplementary Figure 1. Sgk2 ablation had no effect on locomotor activity, food intake, and adipose tissue gene expression during β3 adrenergic stimulation.

(A) Locomotor activity and (B) Cumulative food intake of *Sgk2*$^{+/+}$ (n=8) and *Sgk2*$^{-/-}$ (n=8) male mice prior to and during administration of a β_3AR agonist CL316243. (C, D) Quantitative real-time PCR analysis of genes involved in β_3AR-stimulated brown and beige adipose thermogenesis in *Sgk2*$^{+/+}$ (n=8) and *Sgk2*$^{-/-}$ (n=8) mice treated with CL316243 for 10 days.
